# Optimization of Optomotor Response-based Visual Function Assessment in Mice

**DOI:** 10.1038/s41598-018-27329-w

**Published:** 2018-06-26

**Authors:** Cong Shi, Xuedong Yuan, Karen Chang, Kin-Sang Cho, Xinmin Simon Xie, Dong Feng Chen, Gang Luo

**Affiliations:** 1 Schepens Eye Research Institute, Massachusetts Eye and Ear, Department of Ophthalmology, Harvard Medical School, Boston, MA USA; 2 College of Computer Science, Sichuan University, Chengdu, China; 3 Graduate Institute of Clinical Dentistry, School of Medicine, National Taiwan University, Taipei, Taiwan; 4 AfaSci Research Laboratories, Redwood City, CA USA

## Abstract

Optomotor response/reflex (OMR) assays are emerging as a powerful and versatile tool for phenotypic study and new drug discovery for eye and brain disorders. Yet efficient OMR assessment for visual performance in mice remains a challenge. Existing OMR testing devices for mice require a lengthy procedure and may be subject to bias due to use of artificial criteria. We developed an optimized staircase protocol that utilizes mouse head pausing behavior as a novel indicator for the absence of OMR, to allow rapid and unambiguous vision assessment. It provided a highly sensitive and reliable method that can be easily implemented into automated or manual OMR systems to allow quick and unbiased assessment for visual acuity and contrast sensitivity in mice. The sensitivity and quantitative capacity of the protocol were validated using wild type mice and an inherited mouse model of retinal degeneration – mice carrying rhodopsin deficiency and exhibiting progressive loss of photoreceptors. Our OMR system with this protocol was capable of detecting progressive visual function decline that was closely correlated with the loss of photoreceptors in rhodopsin deficient mice. It provides significant advances over the existing methods in the currently available OMR devices in terms of sensitivity, accuracy and efficiency.

## Introduction

Vision impairment and blindness present an enormous unmet medical need. Visual functions such as visual acuity (VA) and contrast sensitivity (CS) are the gold standard for clinical assessment of vision, and they are widely accepted as outcome measures in drug development. As preclinical studies highly depend on mouse models, there is a particular demand on visual performance assays of small rodents that accurately predict human responses. Behaviorally operant methods have been traditionally employed to assess mouse visual functionalities^1–6^. However, these methods require long-term behavioral training procedures that can take from weeks to months, showing poor scalability for a large number of mice. In contrast, the optokinetic reflex (OKR)^7–12^ or optomotor response (OMR)^13–18^ presents a clinically relevant and readily monitored behavior protocol for visual function tests. OKR/OMR is a stereotyped eye (OKR) or head (OMR) movement in response to movement in the surrounding environment, serving to stabilize the visual image on the retina to allow for high resolution vision. The reflex is highly conserved among vertebrates and does not require a learning process (such as learning to press a lever to get water when seeing a stimulus on the screen in the behaviorally operant methods). OKR/OMR tests have been used to measure VA and CS in rodents by examining eye or head movements tracking rotating stripes.

OMR assay is easier to implement for mice compared to OKR, as it requires no constrains to animals, which are allowed to move freely on the platform. Visual assessment of OMR in unrestrained mice by human observers has been reported, yet the OMR scoring is usually subjective and requires experienced experimenters, since the subtle head movements of mice can be easily missed^13–15^. Recently, semi-automated and fully automated systems for quantifying OMR in unrestrained mice, by examining head movement in responding to rotating stripes, have been developed and rapidly gained popularity^16–18^. Fundamental problems of these OMR systems lie in their crude/inaccurate and lengthy scoring procedures. In part, this is because they were designed to detect only positive OMR indicators (i.e., mouse’s head movement tracking the rotating stripes) without implementation of any negative OMR indicator, which is supposed to explicitly indicate that the mouse was unable to see the stimuli. Prusky *et al*. implicitly concluded that the mouse could not see if the mouse did not show any positive OMR response within an arbitrarily pre-defined time window^14^. In another study, Abdeljalil *et al*. counted the number of positive OMR responses within a time window^14,15^. As these time windows were arbitrarily set and not adjusted to individual mouse under various conditions, these systems were likely to make premature conclusions especially when the mouse was not in compliance or reluctant to look at the stimuli. In the systems developed by Kretschmer *et al*., the frequency data of voluntary head movement obtained from blind mice, i.e. six-month-old *rd1* mice, was used to calculate an “OMR index” and served as references or baselines when positive OMR responses were scored^16–18^. This method essentially ignored the behavioral variability between blind mice and mice with normal vision, as well as the impacts of genetic background, age, sex, and other individual differences on system accuracy.

To advance the automated OMR systems, we propose a novel assessment method that employs mouse head pausing behavior as a negative OMR indicator and an optimized staircase testing protocol. Using both wild-type (WT) and Rhodopsin deficient (*Rho*^*−/−*^) mice which exhibited progressive retinal degeneration^19^, we demonstrated the validity and efficiency of our OMR protocol. Development of an unbiased and rapid OMR assay for assessment of visual functions in mice would greatly facilitate the drug discovery process and enable large scale visual behavior phenotyping in animals.

## Results

### Setup of the OMR system

**Figure 1:**
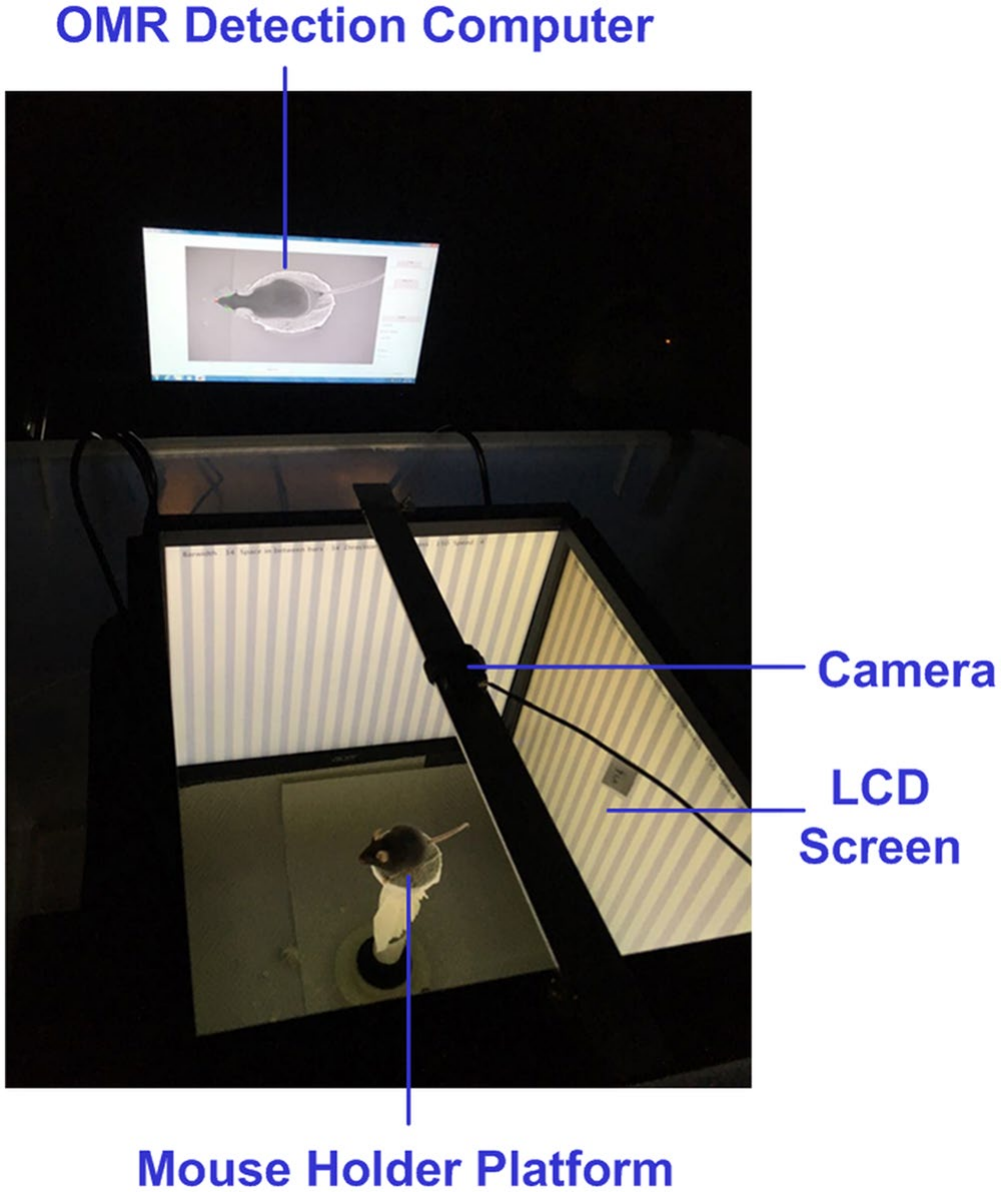
The mouse OMR assessment system. A top-view photograph of the OMR system consisting of (1) a visual stimulus unit which displays rotating black-and-white stripes on 4 LCD screens, (2) a mouse holder platform placed in the middle of the arena, for the mouse to stand and move freely on, and (3) a computer running a house-made OMR detection algorithm to process mouse images captured by a camera mounted above.

**Figure 2:**
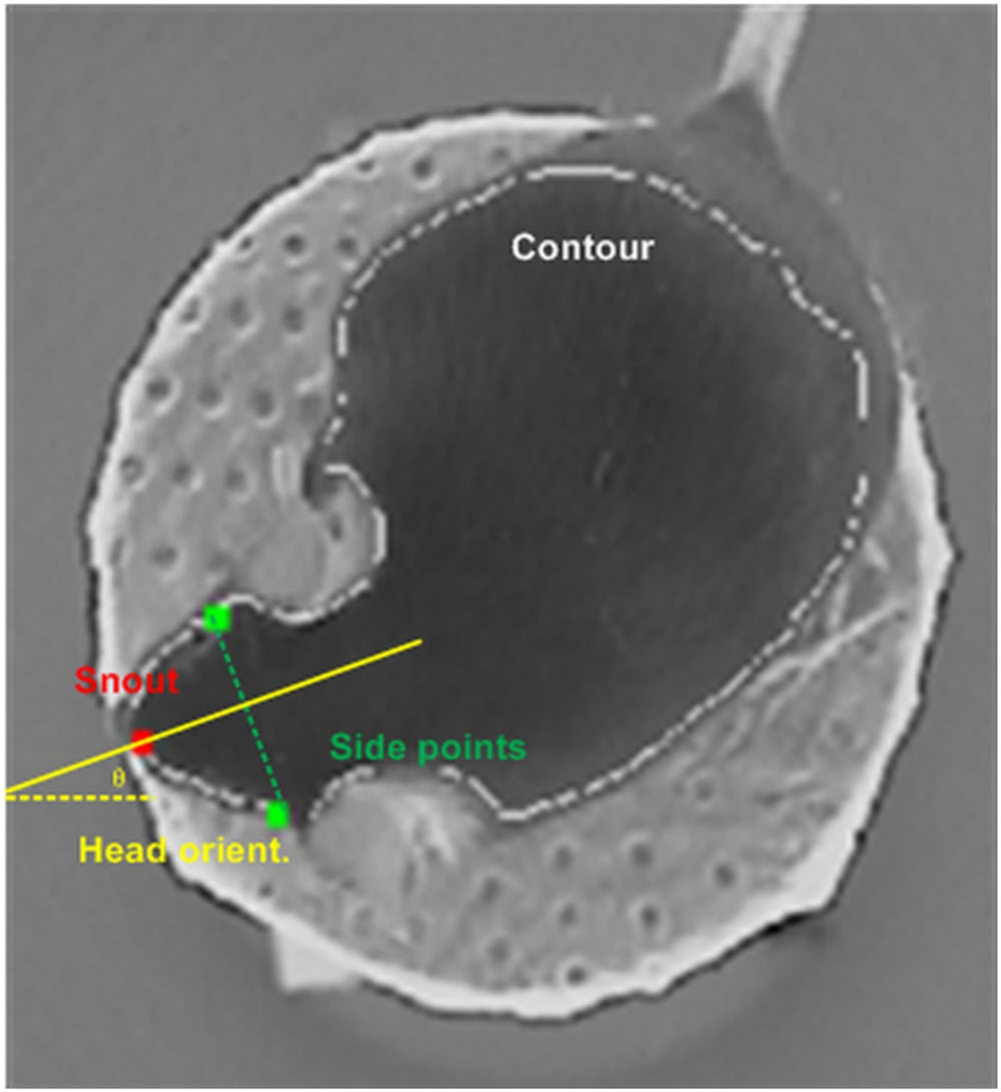
The house-made OMR detection algorithm. A representative screen shot taken from the OMR detection computer. The algorithm flagged positive OMR indicators (head tracking behaviors) and negative OMR indicators (head pausing behaviors) based on the dynamics of the mouse’s head orientation. The head orientation was calculated in three steps: (1) mouse contour extraction, (2) locating the snout as the point with the maximum curvature on the contour, and (3) head orientation computation from the snout point and two side points that have equal distance from the snout along the mouse contour.

To allow OMR assessment in mice, we developed an OMR prototype system as shown in Fig. 1. It is comprised of: (1) a visual stimulus unit to display rotating stripes on 4 LCD screens, (2) a mouse holder platform in the middle of the arena, which allowed the mouse to stand and move freely, and (3) a computer running a house-made OMR detection algorithm (Fig. 2) to process mouse images captured by a camera mounted on top of the platform. See Methods for more details about the OMR system.

**Figure 3:**
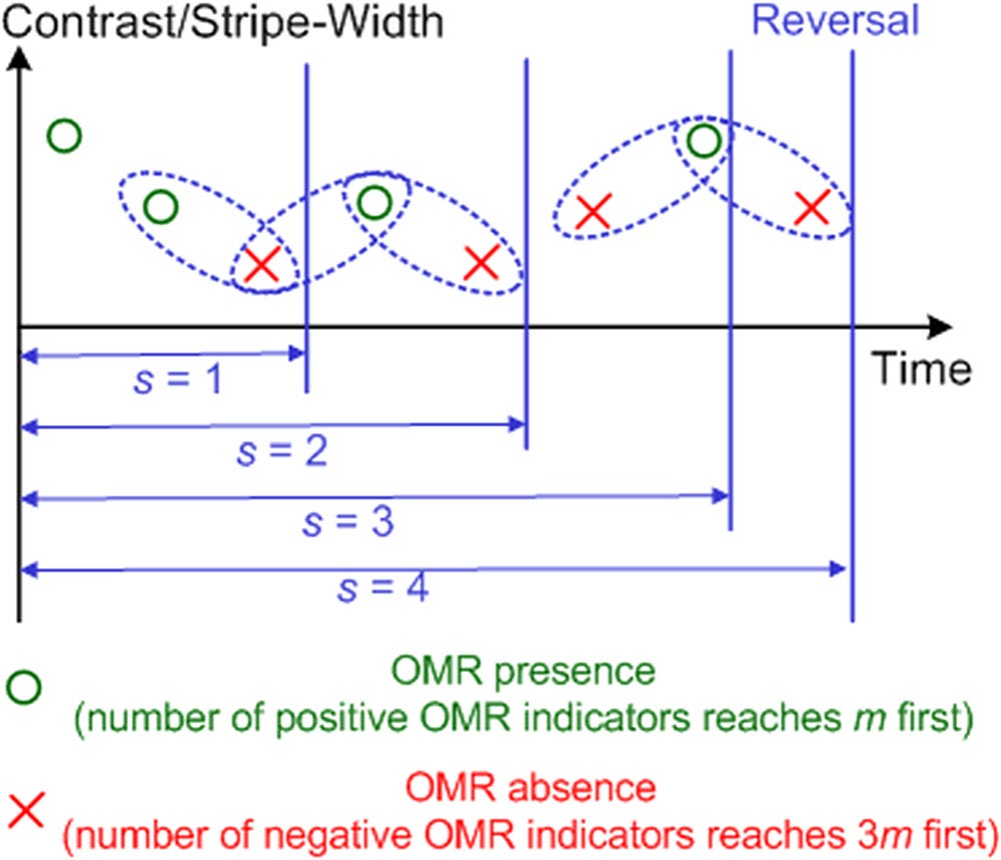
The staircase protocol for OMR tests. This schematic illustrates the staircase protocol for contrast sensitivity (CS) and visual acuity (VA) measurements. The protocol was specified by two parameters: (1) head movement count *m*, and (2) staircase reversal count *s*. If the count of positive OMR indicators (head tracking movements) reached *m* before the count of negative OMR indicators (head pausing status) reached 3 *m*, the protocol asserted an OMR presence (implying that the mouse was able to see the stimuli) and set the stimulus one level harder, i.e. lower contrast (for CS assessment) or higher spatial frequency (for VA assessment). When the count of negative indicators reached 3 *m* before the count of positive ones reached *m*, the protocol asserted an OMR absence (implying that the mouse failed to see the stimuli) and set the stimulus one level easier. The count of trial reversals between one OMR presence and one OMR absence must reach *s* before a conclusive final vision measurement.

A staircase protocol was then implemented for CS and VA assessment by OMR^2,14^. CS is defined as the reciprocal of the lowest contrast perceivable, and VA as the highest spatial frequency perceivable. The protocol was characterized by two parameters: 1) head movement count *m*, and 2) staircase reversal count *s* (See Fig. 3; also refer to the Method section for more details). Our study included two stages: The first stage was aimed to optimize the staircase protocol to allow the achievement of best accuracy with minimal time consumption, using the CS assessment. The second stage validated the feasibility of the optimal protocol by applying it to both CS and VA assessments.

### Protocol optimization

**Figure 4:**
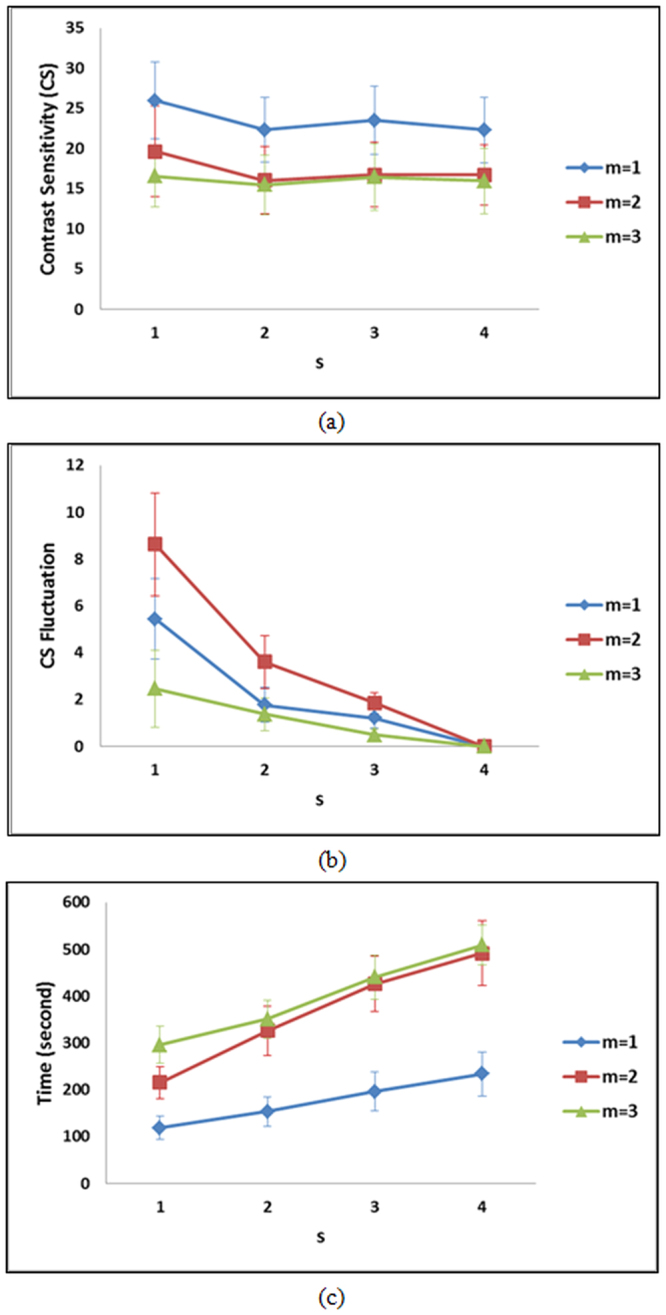
Contrast Sensitivity (CS), CS fluctuation, and time consumption of different staircase protocols. Data were collected from 12 mice, including 9 WTs and 3 *Rho*^*−/−*^. Repeated measures ANOVA tests revealed that varying neither *m* nor *s* caused significant change in the CS value. CS fluctuation for protocol (*m*, *s*) in (**b**) is defined as the absolute difference between the CS measured with (*m*, *s*) and the CS measured with (*m*, 4) (used as reference). CS fluctuation significantly decreased and time consumption significantly increased along with increasing values of *m* and *s*, respectively. Error bars represent S.E.M.

To select an optimal staircase protocol, we tested 12 staircase protocols with different combinations of head movement count *m* (*m* = 1, 2 or 3) and staircase reversal count *s* (*s* = 1, 2, 3 or 4), and compared their measurement reliability and time consumption in 9 WT mice (6~8 week old) and 3 Rhodopsin knockout (*Rho*^*−/−*^) mice (6 week old). A repeated measures ANOVA (three *m* values by four *s* values) revealed that neither *m* nor *s* significantly altered the CS value (*p* = 0.269, *F*_2,20_ = 1.403 for *m*, and *p* = 0.361, *F*_3,30_ = 1.108 for *s*) (Fig. 4a). Although the average CS seemed to be higher when *m* = 1 than when *m* = 2 or 3, a post-hoc test did not find any significant difference between these groups in pairwise comparisons (*p* = 0.660 for *m* = 1 vs 2, and *p* = 0.685 for *m* = 1 vs 3). CS fluctuation for protocol (*m*, *s*), defined as the absolute difference between the CS measured with (*m*, *s*) and the CS measured with (*m*, 4) (used as reference), however, significantly decreased with *m* (*p* = 0.043, *F*_2,20_ = 3.707) and *s* (*p* < 0.001, *F*_3,30_ = 10.457) (Fig. 4b). As expected, time consumption of each test significantly increased along with *m* (*p* = 0.001, *F*_2,20_ = 10.628) and *s* (*p* < 0.001, *F*_3,30_ = 51.462) (Fig. 4c). A much larger increase in time consumption was noted when *m* increased from 1 to 2. In contrast, the time reduction with decreasing *s* was relatively small. The data suggest that while varying either *m* or *s* does not significantly change the CS value, increasing *m* reduces CS fluctuation or improves reliability at a significant cost of time. In contrast, increasing *s* significantly reduces CS fluctuation with a relatively small impact on time consumption.

To select the optimal combination of parameters *m* and *s*, we ranked all the 12 protocols in an ascending order based on CS fluctuation and time consumption, respectively. Each protocol was scored by the sum of its ranks in both orders. The median score was 13.5 and the interquartile range was from 12 to 15. Intuitively, protocols with lower scores would be preferable because they represent small CS fluctuations and short time consumption. Among the 12 protocols, three of them were scored below the interquartile range. They were (*m*, *s*) = (1, 4), (1, 3) and (1, 2), with a score of 6, 8, and 9, respectively. All protocols with *m* = 1 yielded good scores, mainly because of their time saving effects. As the CS fluctuation was not very different among the 3 protocols (0, 1.2, 1.77 for *s* = 4, 3, 2, respectively), *s* = 2 was most favorable because of its larger advantage on shorter time consumption (234s, 196s, 154s for *s* = 4, 3, 2, respectively); therefore, we selected (*m*, *s*) = (1, 2) as the optimal protocol in the following validation experiment.

### Verification of the optimal protocol

**Figure 5:**
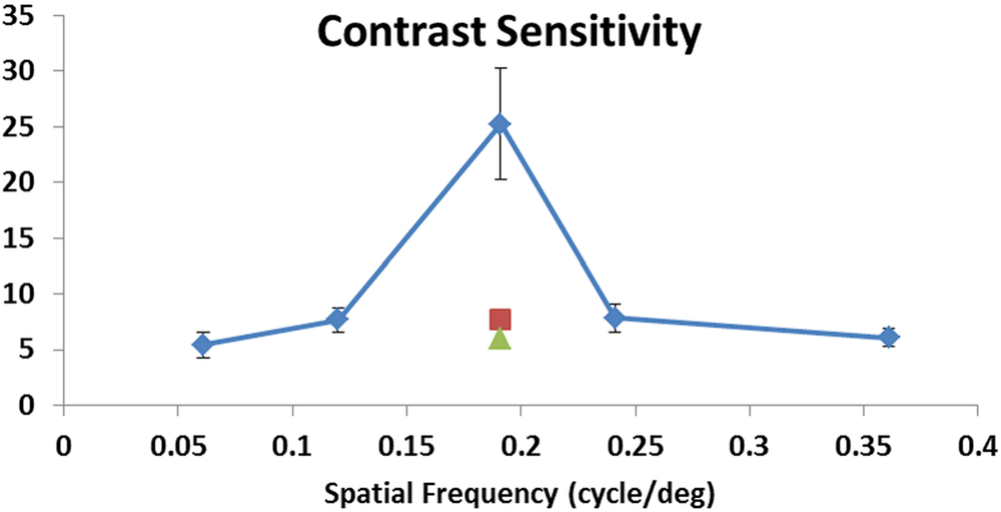
CS assessment in WT mice. CS of WT mice at the age of 6 weeks (*n* = 10) were measured by our OMR system at 5 different spatial frequencies using the optimal protocol. Two CS outliers (labeled as single triangle and square) at the peak spatial frequency of 0.19 cpd were excluded from data analysis, as they were abnormally lower than the two mice’s CS at neighboring frequencies.

We verified the feasibility of the optimal protocol for CS and VA assessments using another 10 WT mice and 11 *Rho*^*−/−*^ mice. We tracked the CS and VA in *Rho*^*−/−*^ mice from the age of 6 weeks onward to follow the visual function changes along the course of their photoreceptor degeneration. WT mice have developed mature and stabilized vision by the age of 6 weeks, while *Rho*^*−/−*^ mice are reported to undergo progressive photoreceptor degeneration from 24 days onward and retinal function abnormality (as determined by electroretinogram) from 7 weeks onward that will eventually lead to blindness by ~4 months old^19^. The progressive loss of photoreceptors and visual functions in *Rho*^*−/−*^ mice are well documented^20^. As the strength of OMR responses is sensitive to spatial frequency, we assessed mouse CS with OMR under varied spatial frequencies. Figure 5 shows that the CS of WT mice at the age of 6 weeks peaked at 0.19 cycles per degree (cpd). Two CS outlier data points (the single triangle and square) at this spatial frequency were excluded from data analysis, because they were abnormally lower than the two mice’s CS values at neighboring frequencies. These results showed higher sensitivity of our protocol in assessing CS in mice than what was previously reported using other methods (see Discussion for details).

**Figure 6:**
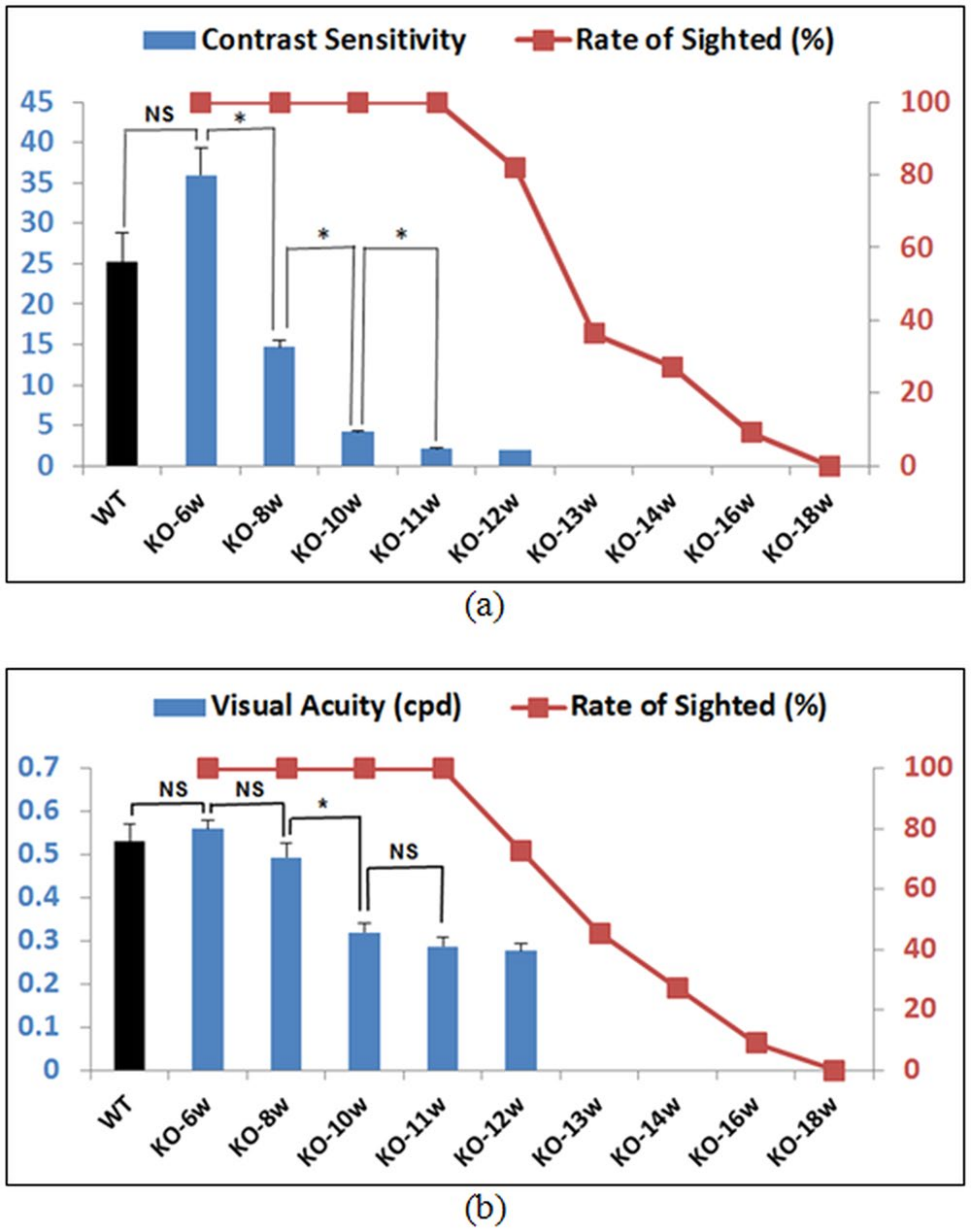
Tracks of CS and VA in WT and *Rho*^*−/−*^ mice of various ages. Peak CS at the spatial frequency of 0.19 cpd (**a**) and VA (**b**) in 6-week-old WT (black bar, *n* = 8) and 6~18 week-old *Rho*^*−/−*^ mice (KO; blue bars, *n* = 11) were measured using the optimal protocol. The rate of sighted (mice) was calculated as the percentage of *Rho*^*−/−*^ mice with measurable CS/VA. Average CS/VA was calculated only for mice with measureable CS/VA, and the average is not reported when the rate of sighted fell below 50%. Error bars represent S.E.M. *Denotes *statistical significance*. NS denotes *no statistical significance*. For other pairs not connected, statistics analyses were not shown for clarity, or could not be performed properly as we stopped tracking completely blind mice.

We next tracked the change of peak CS value (measured at the *peak* spatial frequency of 0.19 cpd) in *Rho*^*−/−*^ mice from 6 weeks onward. The average peak CS value of WT and *Rho*^*−/−*^ mice at 6 weeks showed no significant difference, as shown in Fig. 6a (25.2 for WT and 35.9 for *Rho*^*−/−*^, *p* = 0.10, t-test). As expected, the peak CS value of *Rho*^*−/−*^ mice dropped gradually after the age of 6 weeks; by the age of 8 weeks, it had already dropped substantially to ~15. The rate of sighted mice, which is defined as the percentage of mice with a measurable CS, was calculated. We noted that while all *Rho*^*−/−*^ mice had detectable OMR by the age of 10 weeks, over half of *Rho*^*−/−*^ mice showed no measureable CS by the age of 13 weeks, suggesting that half of these mice were blind. Average peak CS was calculated only for mice with measureable CS, and the average was not reported when the rate of sighted fell below 50% in Fig. 6a.

We then assessed VA in WT and *Rho*^*−/−*^ mice using the optimized protocol. The VA of *Rho*^*−/−*^ mice at the age of 6 weeks were comparable with that of WT mice (0.53 cpd for WT and 0.56 cpd for *Rho*^*−/−*^, *p* = 0.665, t-test), and it also started to drop from 8 weeks onward, albeit not as drastically as seen in the CS assessment (Fig. 6b). The rate of sighted mice, as calculated by the percentage of mice with a measurable VA, was about the same as that obtained using the CS assessment in Fig. 6a. By the age of 13 weeks, more than half of *Rho*^*−/−*^ mice showed no measureable VA. By the age of 18 weeks, no *Rho*^*−/−*^ mice showed measurable VA, or in other words, they all became blind. This rate-of-sighted curve measured by VA matches perfectly with that measured by CS assessment. Similarly, the average VA was not reported when the rate of sighted fell below 50% in Fig. 6b.

## Discussion

In this study, we employed a novel negative OMR indicator, mouse head pausing status, to replace the arbitrarily pre-set time window or blind mouse’s OMR baseline as used in previous studies^14–18^. When this negative indicator was applied in combination with a positive OMR indicator (the head tracking state in response to visual stimuli), we were able to implement an unambiguous, fast staircase protocol to assess visual functions in WT and *Rho*^*−/−*^ mice. We showed that the staircase protocol characterized by head movement count *m* = 1 and staircase reversal count *s* = 2 is an optimal setting considering accuracy and efficiency for vision function assessments. We showed that this optimal protocol enables quick measurements of visual functions in mice with normal or impaired vision. The entire procedure required less than 3 minutes per mouse (Fig. 4c). No mice were trained or hand-picked by any means, while this was usually done in operant studies. The protocol can be easily implemented into a completely automated system.

Our results of CS, VA, and peak spatial frequency of stimuli at which peak CS or equivalent response was obtained are in agreement with those in the literature (Table 1). Our measured VA (0.53 cpd) and peak frequency (0.19 cpd) are within the reported range (0.4 ~ 0.6 cpd and 0.1 ~ 0.2 cpd, respectively). Importantly, our CS results (25 for WT mice and 35 for 6-week-old *Rho*^*−/−*^ mice) are at the high end of the reported CS range, suggesting a high sensitivity of our protocol. Only two papers reported CS value higher than ours. First, Histed *et al*. reported CS of 50 from the 3 most well-behaved animals they hand-picked from 21 mice^1^. It is possible that their finding was biased towards animals with a better vision. In contrast, all of the 33 mice tested in our experiments were randomly picked and shipped directly from Jackson Laboratory without any filtering process, thus they included individual variability. Some mice in our study indeed showed a CS of 50. Second, Van Alphen *et al*. also reported an extremely high CS of 100 and peaked at 0.17 cpd^9^. As this is by far the only study reporting such a high CS level, which is about the same as human eyes^21^, the finding may need further confirmation. Overall, our method was shown to be able to measure mouse CS with a high sensitivity.

**Table 1:** Summary of literature reports on mouse CS, VA and peak spatial frequency values.

No.	Work	Method	Subjects	CS	VA (cpd)	Peak freq. (cpd)
1	Gianfranceschi *et al*.^2^	Operant	4 WT (C57BL/6J)	—	0.5	0.15
2	Prusky *et al*.^5^	Operant	5 WT (C57BL/6)	6	0.55	0.2
3	Busse *et al*.^6^	Operant	7 WT (C57BL/6J) + 5 *trans* (2 HHrtTAXK and 3 TRE/ASTBDN-1)	5~10	—	0.13
4	Histed *et al*.^1^	Operant	3 WT (C57BL/6-Balb/c hybrid)	50	0.6	0.05~0.1
5	Sinex *et al*.^7^	OKR	6 WT (C57BL/6J)	—	0.5	0.125
6	Tabata *et al*.^10^	OKR	14 WT (C57BL/6J)	7	0.5	0.125
7	Benkner *et al*.^12^	OKR	12 WT (C57BL/6)	12.5	0.4	0.15
			4 *rd10* @6wk	5	0.35	0.15
8	van Alphen *et al*.^9^	OKR	15 WT (C57BL/6)	100	0.5	0.17
9	Kretschmer *et al*.^18^	OKR	10 WT (C57BL/6J)	20	0.4	0.15~0.2
		OMR	7 WT (C57BL/6J)			
10	Kretschmer *et al*.^16^	OMR	6 WT (C57BL/6J)	—	0.4	0.2
11	Prusky *et al*.^14^	OMR	11 WT (C57BL/6)	24.5	—	0.064
			17 WT (C57BL/6)	—	0.4	—
12	Abdeljalil *et al*.^15^	OMR	10 WT (C57BL/6J)	—	0.52	0.26
13	Ours	OMR	10 WT (C57BL/6J)	25	0.53	0.19
			11 *Rho*^*−/−*^@6wk	35	0.56	—

As summarized in Table 1, our VA results were similar to findings using operant methods. In general, many of the previous OMR methods reported a lower VA (except that by van Alphen *et al*., who also reported an extremely high CS value^9^) than the operant methods. We believe that this may be due to the lack of a precise negative OMR indicator. As Kretschmer *et al*. showed, mouse’s OMR responses become less obvious as visual stimuli are weaker (i.e. smaller OMR gain)^16,18^. When the stimulus approaches to the visual threshold, the weak OMR may be mixed with many other voluntary head movements, thereby leading to premature conclusion of a visual threshold. Implementation of negative OMR indicators ignoring voluntary head movements allowed us to apply a staircase protocol to accurately pinpoint the visual thresholds. Since visual function assessment is about finding the threshold, staircase testing has inherent advantages over other methods relying on arbitrary baselines, by focusing on the visual thresholds to acquire more accurate and reliable results. Moreover, focusing on thresholds greatly saves testing time on easily visible stimuli.

Our results are also in line with those acquired using the electrophysiological methods. For example, VEP has been used to evaluate visual physiology that has a counterpart in visual behavior, including VA and CS, in mice^22–25^. Previous work of VEP recording in conscious mice reported the VA (0.60 ± 0.15 and 0.62 ± 0.156 cpd, respectively) similar to that of ours in normal C57BL/6J mice^26,27^.

Presently, we do not yet have a definite explanation for the physiological mechanism of the head pausing behavior. We speculate that it may implicate that the mouse is attending to the screen, similarly to meerkats watching for predators. Since OMR is an involuntary behavior when mouse sees the stimuli, head pausing might suggest that the mouse tries to look but cannot detect the moving stimuli. While Kretschmer *et al*. used random and voluntary head movements of mice to calculate OMR baseline^16,18^, we argue that this is inappropriate to be taken into consideration for vision assessment. For instance, rodent head bobbing (swaying head from side to side) is believed to be a behavior that allows the animal to acquire depth perception, because such movement can create visual parallax^28,29^. An unstill head can also be a sign of animal incompliance, or environment exploring with other sensory modules.

Our study suggests that CS is a more sensitive indicator for vision loss than VA in *Rho*^*−/−*^ mice, especially at the early stage of retinal degeneration. From ages of 6 to 8 weeks, CS in *Rho*^*−/−*^ mice dropped from 36 to 15 (Fig. 6a), an average 59% reduction, while their VA dropped only from 0.56 to 0.49 cpd (Fig. 6b), merely 12.5% reduction on average. This result is in line with the findings from clinical studies in human patients^30–32^. Kleiner *et al*. found that CS is useful for measuring visual loss not detected by standard Snellen charts in patients with macular degeneration^33^. These data suggest that measuring CS in animal models is highly relevant to preclinical research.

While *Rho*^*−/−*^ mice do not have rod function since birth, their cone degeneration typically starts from postnatal week 7, as electrophysiology study by Jaissle *et al*. showed^34^. They also showed that “no clear ERG signal was detectable after postnatal week 13 under photopic conditions”. Our data are consistent with their findings. The time course of photopic vision loss in *Rho*^*−/−*^ mice is similar to human beings with retinitis pigmentosa, who first develop night blindness followed by central vision loss or even complete blindness. Therefore, using the *Rho*^*−/−*^ model to evaluate measurement of CS and VA under photopic conditions is appropriate.

Our study included non-hand-picked mouse subjects (total *n* = 33 in both experimental stages, including WT as well as *Rho*^*−/−*^ mice), and followed up on the visual functions of 11 *Rho*^*−/−*^ mice from 6 to 18 weeks in their postnatal lives. We observed large individual variations in visual functions among the WT as well as *Rho*^*−/−*^ mice. Some *Rho*^*−/−*^ mice became blind at the age of 12 weeks, while other showed measureable vision until the age of 18 weeks. At the age of 6 weeks, there was a wide range of CS (from 10 to 50) in our sample. Such large between-group variabilities underscore the importance of within-group study designs, in which individual differences can be canceled by each individual subject and therefore statistical power can be achieved with relatively small sample sizes. By measuring mice efficiently over long time course without many compromises due to, for example, training, stress, and anesthesia, we have demonstrated the potential of our optimized method for medical research involving small animals.

This OMR system can be extended to other mouse models in the future. For instance, OMR assessments in mice with various retinal cell defects, including primary cone pathology and/or ganglion cell pathology, may be valuable. Also, implementation of an infrared image camera could allow evaluation of mouse head movements under a dim light. Our current OMR detection algorithm is effective for animals of other colors, as long as a good color contrast between the mouse skin color and the background of the platform is provided. To offer a proof of concept, we performed a small scale experiment that assessed CS and VA in white colored Balb/c mice (6-weeks-old, *n* = 5). Like what was reported by other researchers^35^, we found Balb/c mice responded to the rotating stripes by moving their head toward an opposite direction of stimuli. We thus reconfigured the OMR detection program to track head motion against the direction of the rotating stimuli. Our detected average CS was 3.078 ± 0.720, and VA was 0.476 cpd ± 0.063 from these mice (Supplementary Fig. 1). The low CS value of Balb/c mice was similar to that reported by Yeritsyan *et al*. using an OMR method (CS = 2.2)^35^. However, we detected much higher VA in Balb/c mice than their OMR result, 0.12 cpd. Interestingly, when using the visual water task (an operant method), Yeritsyan also detected much higher VA in Balb/c mice (0.3 cpd) than that acquired using their OMR method^35^. Their data suggested that without implementing a negative OMR indicator, OMR assessment was likely to yield a lower reading in mice due to premature ending of the test. Together, our results demonstrated sensitive and consistent readout of CS and VA assessments in various mouse lines using the present automated OMR system.

## Methods

### OMR system

For automated and unbiased assessment of visual behavior in mice, we built an OMR prototype system, which was comprised of three units, as shown in Fig. 1. (1) A visual stimulus unit with four LCD screens (Acer 15′′) was used for displaying rotating black-and-white stripes. The luminance of the screen was calibrated using a luminance meter (Minolta LS-100). The width, direction and contrast of stripes were configurable and manually set during the experiments depending on the mouse’s response. (2) A platform was set in the middle of the arena for mouse to stand and move freely on. (3) A computer vision unit based on a desktop computer (Intel i7-4790 CPU @ 3.60 GHz) was running a house-made OMR detection algorithm to process the real-time mouse images captured by a 30 frames/sec (fps) camera mounted on top of the platform. The algorithm extracted and smoothed the contour of the mouse body, and then located the mouse snout as the maximum curvature point on the contour (Fig. 2). Two head-side points with equal distance to the snout point along the contour were also identified. The distance was a fixed fraction with respect to the length of the mouse contour. The mouse head orientation (*θ* in Fig. 2) was computed as the orientation of the line that connected the snout point and the mid-point of the two head-side points (The yellow line in Fig. 2). The snout coordinate trajectory was also recorded. Signals of head orientations and snout coordinate trajectory were filtered to remove motion noises (such as abrupt mouse head jitters lasting less than two frames, or the small-amplitude vibration of the snout points due to non-uniform luminance conditions, etc.) using a 3-point medium filter.

The algorithm performed OMR detection based on the mouse head orientations and snout trajectory in the latest 20 frames (equivalent to 0.67 second with the 30 fps camera). When a new image frame was captured, the mouse head was regarded to be in a tracking state and a positive OMR indicator was recorded if the following three conditions were simultaneously met: (1) the displacement of mouse snout between any two successive frames in the latest 20 frame time was below a threshold *d* (meaning no large motions); (2) the accumulated head rotation (measured as unwrapped head orientation difference between the first and the last of the latest 20 frames) exceeded an angular threshold *α* in the direction of rotating stripes; (3) the accumulated snout movement (measured as the distance between snout coordinates of the first and the last of the latest 20 frames) was over a small distant threshold *c*. In contrast, the mouse head was regarded as to be in a pausing state and a negative OMR indicator was recorded if the following two conditions were simultaneously met: (1) the displacement of mouse snout between any two successive frames in the latest 20 frame time was below *d*; (2) the accumulated snout movement was below *c*/2 (regardless of the accumulated head rotation). Those thresholds are configurable to match various mouse sizes. Once either a positive or negative OMR indicator was recorded, the OMR detection was suspended during the next 19 frames to avoid repeated detections (but mouse head orientation and the snout trajectory were still recorded). Consecutive recordings of negative OMR indicators (such as the negative indicators detected at the 20th, 40th and 60th frames) were merged into one to further remove repeated OMR absence detections, as pausing status usually lasted for a longer time. However, consecutive positive OMR indicators were not merged, as occasional long-time head tracking behaviors were strong evidence that the mouse could see the rotating stripes.

In our prototype system, the stripe patterns were manually changed. The stimuli were always on until the presence or absence of OMR was confirmed, and the stimuli parameters, such as contrast or spatial frequency, were then changed manually. We have manually checked the CS and VA values obtained using either the constant display or the short on-off display and found no obvious difference between these two methods. OMR was clearly observed in mice exposed to constant display (or short display) of stimuli throughout the experiment.

To ensure experimenters engaged in the experiment procedure to confirm that the protocol is reasonable, we did not implement an automated configuration of the stimulus pattern. A fully-automated OMR system will be developed in the future.

### Staircase protocol of OMR tests

Since many mice would get agitated when they came to the testing platform for the first time, we pre-trained the mice by sitting them in the OMR system with rotating strips for ~10 minutes the day before the OMR test so the mice were adapted to the system. Such acclimatization is a common practice in behavior studies. To measure visual functions including contrast sensitivity (CS) and visual acuity (VA), we implemented the 1-up-1-down staircase protocol (Fig. 3) for our OMR detection algorithm. Michelson contrast definition was used in this study, and CS is the reciprocal of the lowest contrast perceivable by mice. VA was reported as the highest spatial frequency perceivable (in cpd) with highest contrast available in the test. The smallest CS step was set as 0.063, and the smallest VA step as 0.050 cpd (~8% of the VA range from 0.06 to 0.72 cpd). Comparing to Snellen vision charts designed for human beings, which grade human VA by 11 levels from 20/200 to 20/10 with smallest step of 3 cpd (10% of the range), the VA step in our study was reasonably small.

The protocol was specified by two parameters: head movement count *m* and staircase reversal count *s*. If the count of positive OMR indicators (head tracking movements) reached *m* before the count of negative OMR indicators (head pausing status) reached 3 *m*, the protocol asserted an OMR presence (implying that the mouse was able to see the stimuli) and set the stimulus one level harder, i.e. lower contrast (for CS assessment) or higher spatial frequency (for VA assessment). When the count of negative indicators reached 3 *m* before the count of positive ones reached *m*, the protocol asserted an OMR absence (implying that the mouse failed to see the stimuli) and set the stimulus one level easier. The count of trial reversals between one OMR presence and one OMR absence must reach *s* before a conclusive final vision measurement.

Our study included two stages. The first stage aimed to choose an optimal staircase protocol, and the second stage validates the application feasibility of the optimal protocol. We selected the optimal protocol from 12 possible candidates with different combinations of head movement count *m* (*m* = 1, 2 or 3) and staircase reversal count *s* (*s* = 1, 2, 3 or 4). Each protocol candidate was evaluated on three merits: CS, CS fluctuation, and time consumption for the measurement. The CS fluctuation for protocol (*m*, *s*) was defined as

1 $$CS\_Fluctuation(m,s)=|CS(m,s)-CS(m,4)|.$$

Here we used the measured CS with *s* = 4 as a reference to compute the fluctuation, since empirically more reversals generate more converged and thus more accurate test results (unless the subject is too tired to continue the test).

### Animals

All animal procedures were performed in accordance with the statement of the Association for Research in Vision and Ophthalmology, and the protocols were approved by Institutional Animal Care Committee (IACUC) of the Schepens Eye Research Institute. Mice were housed in a temperature-controlled room with a 12 h light/dark cycle. Fresh water and rodent diet were available at all times. Adult C57BL/6J wild-type (WT) and Balb/c mice were purchased from Jackson Laboratory, and mice deficient for Rhodopsin (*Rho*^*−/−*^) were a gift from M. Young’s Lab at Schepens that was originally developed by Humphries and his team^19^, and were bred at the animal facility of the Schepens Eye Research Institute. Mouse genotype was verified by polymerase chain reaction (PCR) for tail DNA as previously described^36^.

### Statistical analysis

To select the optimal staircase protocol, we compared the impact of choosing different *m* and *s* values on measured CS, CS fluctuation, and consumed test time, as mentioned above. SPSS (version 11.5) was used to analyze the two protocol parameters. We first checked the normality of the CS data for the 9 WT and 3 *Rho*^*−/−*^ mice, grouped under each of the 12 different combinations of *m* and *s* values, and found the data were not significantly different from the normal distribution. Parametric methods, repeated measures ANOVA, and paired T-tests, were used in the statistical analyses. A *p* value smaller than 0.05 was regarded as statistical significance.

## Electronic supplementary material

Supplementary Figure 1
