# Supplementary material for: Optimization of Optomotor Response-based Visual Function Assessment in Mice
Source: Sci Rep. 2018 Jun 26;8:9708. doi: 10.1038/s41598-018-27329-w (PMC6018764; doi:10.1038/s41598-018-27329-w)
Supplement: Supplementary file 1 — Supplementary Figure 1 [file 41598_2018_27329_MOESM1_ESM.docx]

**Optimization of Optomotor Response-based Visual Function Assessment in Mice**

Cong Shi^1†^(e-mail: cong_shi@meei.harvard.edu) (corresponding author),

Xuedong Yuan^2†^ (e-mail: yxd@scu.edu.cn),

Karen Chang^1,3^ (e-mail: karen_chang@meei.harvard.edu),

Kin-Sang Cho^1^ (e-mail: kinsang_cho@meei.harvard.edu),

Xinmin Simon Xie^4^(e-mail: simonxie@afasci.com),

Dong Feng Chen^1*^(e-mail: dongfeng_chen@meei.harvard.edu),

Gang Luo^1*^(e-mail: gang_luo@meei.harvard.edu)

^1^Schepens Eye Research Institute, Massachusetts Eye and Ear, Department of Ophthalmology, Harvard Medical School, Boston MA, USA

^2^College of Computer Science, Sichuan University, Chengdu, China

^3^Graduate Institute of Clinical Dentistry, School of Medicine, National Taiwan University, Taiwan

^4^AfaSci Research Laboratories, Redwood City, CA, USA

† * Made equal contributions


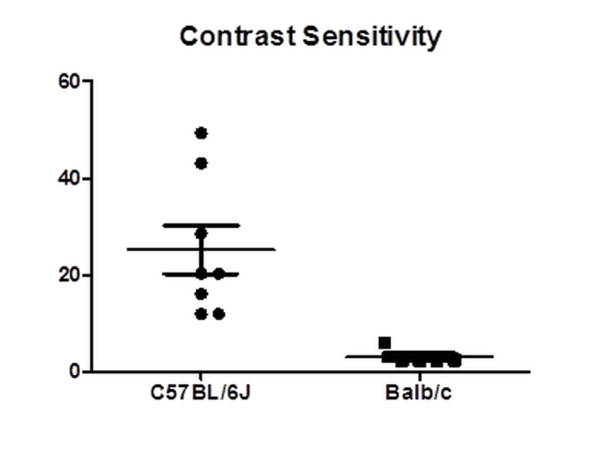

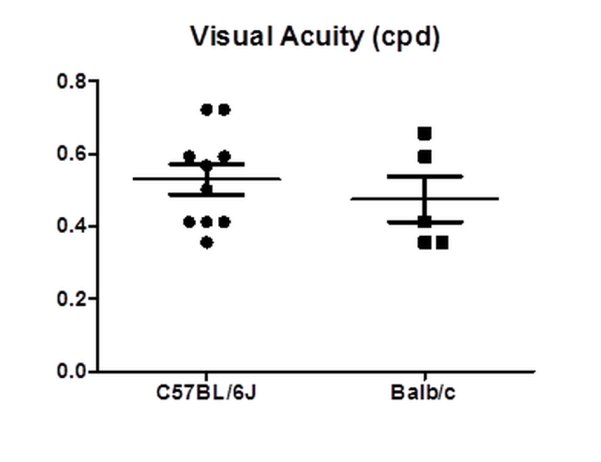


(a) (b)

**Supplementary Figure 1. CS and VA of C57BL/6J and Balb/c mice.** (a) CS of 6-week-old C57BL/6J mice (*n* = 8, two outliers are not included as mentioned in Figure 5) were significantly higher (*p* = 0.003) than that of 6-week-old Balb/c mice (*n* = 5). (b) VA of C57BL/6J mice were similar to that of Balb/c mice (*p* = 0.493). Value = mean ± SEM.
